# Problem Solving as Probabilistic Inference with Subgoaling: Explaining Human Successes and Pitfalls in the Tower of Hanoi

**DOI:** 10.1371/journal.pcbi.1004864

**Published:** 2016-04-13

**Authors:** Francesco Donnarumma, Domenico Maisto, Giovanni Pezzulo

**Affiliations:** 1 Institute of Cognitive Sciences and Technologies, National Research Council, Rome, Italy; 2 Institute for High Performance Computing and Networking, National Research Council, Naples, Italy; Indiana University, UNITED STATES

## Abstract

How do humans and other animals face novel problems for which predefined solutions are not available? Human problem solving links to flexible reasoning and inference rather than to slow trial-and-error learning. It has received considerable attention since the early days of cognitive science, giving rise to well known cognitive architectures such as SOAR and ACT-R, but its computational and brain mechanisms remain incompletely known. Furthermore, it is still unclear whether problem solving is a “specialized” domain or module of cognition, in the sense that it requires computations that are fundamentally different from those supporting perception and action systems. Here we advance a novel view of human problem solving as *probabilistic inference with subgoaling*. In this perspective, key insights from cognitive architectures are retained such as the importance of using subgoals to split problems into subproblems. However, here the underlying computations use probabilistic inference methods analogous to those that are increasingly popular in the study of perception and action systems. To test our model we focus on the widely used Tower of Hanoi (ToH) task, and show that our proposed method can reproduce characteristic idiosyncrasies of human problem solvers: their sensitivity to the “community structure” of the ToH and their difficulties in executing so-called “counterintuitive” movements. Our analysis reveals that subgoals have two key roles in probabilistic inference and problem solving. First, prior beliefs on (likely) useful subgoals carve the problem space and define an implicit metric for the problem at hand—a metric to which humans are sensitive. Second, subgoals are used as *waypoints* in the probabilistic problem solving inference and permit to find effective solutions that, when unavailable, lead to problem solving deficits. Our study thus suggests that a probabilistic inference scheme enhanced with subgoals provides a comprehensive framework to study problem solving and its deficits.

## Author Summary

How humans solve challenging problems such as the Tower of Hanoi (ToH) or related puzzles is still largely unknown. Here we advance a computational model that uses the same probabilistic inference methods as those that are increasingly popular in the study of perception and action systems, thus making the point that problem solving does not need to be a specialized module or domain of cognition, but it can use the same computations underlying sensorimotor behavior. Crucially, we augment the probabilistic inference methods with *subgoaling* mechanisms that essentially permit to split the problem space into more manageable subparts, which are easier to solve. We show that our computational model can correctly reproduce important characteristics (and pitfalls) of human problem solving, including the sensitivity to the “community structure” of the ToH and the difficulty of executing so-called “counterintuitive” movements that require to (temporarily) move away from the final goal to successively achieve it.

## Introduction

Problem solving consists in finding efficient solutions to novel tasks for which predefined solutions are not available [1]. Humans and other animals can efficiently solve complex problems [2, 3] but the underlying neuronal and computational principles are incompletely known. Research on the neuronal underpinnings of problem solving has often proceeded in two different ways. First, researchers have focused on how individual brain areas or circuits solve problems in specific domains; for example, the hippocampus is considered to be implied in solving navigation problems [4–6] and parieto-frontal regions are considered to be implied in mathematical problem solving [7]. This approach is compatible with the idea that the brain has dedicated neuronal machinery to solve domain-specific problems, with little hope to find common principles across them.

A second line of research has focused on *domain-general* problem solving strategies, as exemplified in the realization of *general problem solvers* and other influential cognitive architectures in cognitive science [1, 8–13], planners and problem solvers in AI [14–16], and the recent view of the brain as a statistical engine [17–19]. A challenge in this second research line is to identify core computational principles of planning and problem solving that are, on the one hand, valid across multiple cognitive domains (e.g., sensorimotor tasks, navigation, and mathematical problem solving) and, on the other hand, can be implemented in neuronal hardware and work well in ecologically valid contexts [20].

In this article we show that problem solving can be characterized within a *probabilistic inference* framework. This framework is increasingly used across multiple domains (sensorimotor [21, 22], decision-making and planning [23–25], human-level reasoning [26–28] and learning [29]) and levels of description (higher / computational and lower / neuronal [17, 18, 30–33]), supporting the idea that problem solving does not necessarily require specialized mechanisms that are distinct from those used by perception and action systems.

Our problem solving approach is framed within the *planning-as-inference (PAI)* framework, which casts planning as a probabilistic inference problem [23, 34–38]. In this perspective, goals are “clamped” (i.e., they are treated as “future observations” that the system strives to achieve) and probabilistic inference permits to select the sequence of actions that fills the gap between current and goal states. Despite its usefulness to explain goal-directed behavior [25, 39–41] and to design robot architectures [42], the standard PAI framework fails to capture some important aspects of (human) problem solving, such as the ability to exploit the “junctions” of problems and to subdivide them into more manageable subproblems.

Here, in keeping with a long tradition in human problem solving and cognitive architectures, we augment the PAI approach with a *subgoaling* mechanism that permits splitting the original problem into more manageable, smaller tasks whose achievement corresponds to milestones or subgoals of the original problem [1]. For example, navigation problems can be decomposed by using subgoals (e.g., landmarks) such as “reach the Colosseum, then reach the Imperial Forum” (if one lives in Rome) and puzzles like the Tower of Hanoi can be decomposed by using subgoals such as “free up third rod”.

The importance of subgoaling has been widely recognized by the most popular architectures for symbolic problem solving [1, 8, 10, 13] and in other domains such as connectionist networks, hierarchical reinforcement learning, AI, planning, and robotics [14–16, 35, 43–48]. However, opinions differ about the mechanisms underlying subgoaling. Most systems, especially within the human problem solving and AI traditions [1, 13], assume that subgoaling proceeds *backward* from the final goal states and serves to resolve “impasses”: if a goal-achieving action cannot be executed because of a missing precondition, achieving the precondition becomes the next system subgoal; and so on.

Instead, converging evidence from empirical studies highlights the importance of *feedforward* mechanisms for subgoaling and “search” (i.e., mechanisms that proceed from the current to the goal state) in living organisms. The importance of feedforward mechanisms emerges from psychological experiments [49, 50] as well as neurophysiological studies on rodents [51–53], monkeys, [54, 55] and humans [56].

In keeping, we implement subgoaling within a feedforward probabilistic inference (PAI) scheme, showing that the proposed method reproduces characteristic signatures of human problem solving. To this aim, we present four simulation experiments in the widely-used Tower of Hanoi (ToH) task [57]. We show that in our simulations successes and failures of solving the ToH (e.g., the failure of dealing with *counterintuitive movements*) correspond to the successful identification or a misidentification of subgoals during the inference, respectively.

## Methods

### Formal approach to the Tower of Hanoi (ToH) task

**Fig 1:**
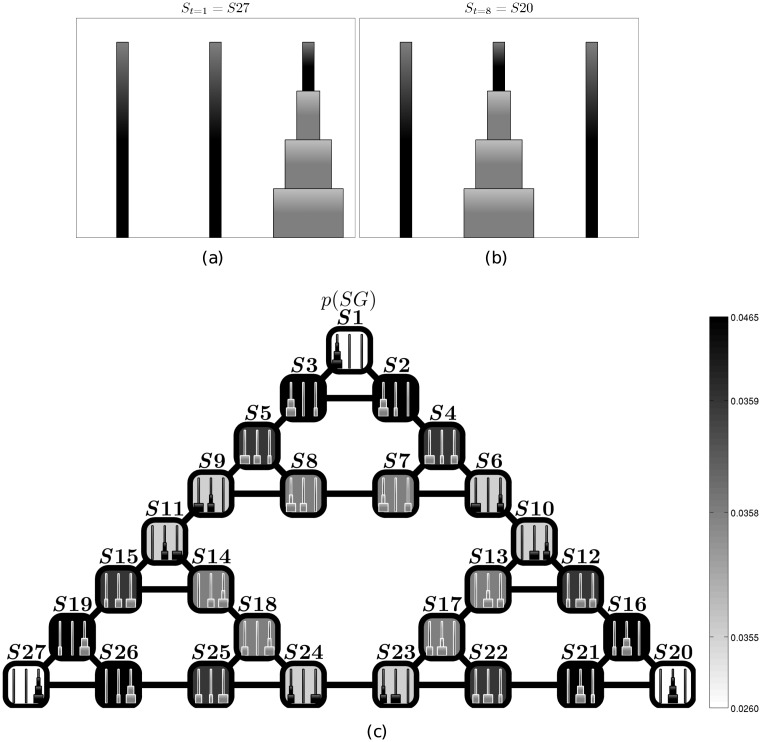
The Tower of Hanoi (ToH) setup. Sample initial (a) and goal (b) position of a Tower of Hanoi problem. (c) Mapping of the Tower of Hanoi problem into an equivalent path-planning problem with 27 states. The levels of grey correspond to the priors on the *SG* distribution; note that the way the priors are calculated is explained in the section on “Subgoal a priori distribution”. In this example, the states have the following values: 0.026 for *S*1, *S*20, *S*27; 0.0355 for *S*6, *S*9, *S*10, *S*11, *S*23, *S*24; 0.0358 for *S*7, *S*8, *S*13, *S*14, *S*17, *S*18; 0.0359 for *S*4, *S*5, *S*12, *S*15, *S*22, *S*25; 0.0465 for *S*2, *S*3, *S*16, *S*19, *S*21, *S*26. For the sake of clarity, the insets (of the states) illustrate the equivalent position in the Tower of Hanoi. The problem consisting in going from the starting position shown in panel (a) to the goal position shown in panel (b) is equivalent to the problem of finding a path from state *S*27 to state *S*20 in panel (c).

The Tower of Hanoi (ToH) task has been widely used in neuropsychology to study executive function and deficits in planning [57, 58]. A standard ToH consists of three disks having different sizes that can slide into three rods to form goal configurations. The aim of the game is starting from any initial configuration of disks and reach a goal configuration using the smallest number of actions. Fig 1 shows sample initial (a) and goal (b) configurations of a ToH. The rules of the game prescribe that only one disk can be moved at a time and no disk may be placed on top of a smaller disk. The quality of the solutions found by subjects performing a ToH puzzle is usually described using the number of movements required to achieve the goal configuration and the reaction time [57].

To model the Tower of Hanoi task, here we use a standard approach in AI [59] that consists in mapping the original task into a path-planning problem, whose states and transitions are shown in Fig 1C. The resulting problem has 27 states (squares) and 39 transitions (edges).

To solve ToH problems (e.g., go from S27 to S20), we use a feedforward probabilistic inference method that iteratively samples candidate subgoals from a prior distribution and uses them as waypoints until a complete solution is found. Specifically, our methods use and extend the *planning as probabilistic inference (PAI)* framework, in which planning is cast as a probabilistic inference problem [23, 25, 34, 37, 40, 60]. In this perspective, a planning strategy can be attained by imposing (“clamping”) goal or rewarding states as (future) observations, so as to bias the probability of future events towards such desirable states.

In problems like the ToH having moderately large state spaces, exploring all the possible paths is very demanding (in some cases, computationally intractable). For example, despite the limited number of states and transitions of the Tower of Hanoi task shown in Fig 1C, this problem allows for about ∼10^12^ possible policies, i.e., mappings from states to actions [61]. In this case, a mechanism for splitting the problem into more manageable subproblems—like subgoaling—is helpful.

In keeping, our method differs from the standard PAI approach because we augment the probabilistic scheme with a *subgoaling* mechanism that permits to split the original problem into smaller (and less complex) subproblems. A second difference with the standard PAI approach is that (akin to Active Inference theory [30]) we do not treat goals as future observations but as states having high (Bayesian) priors.

The rest of this Section introduces the key components of our approach. It is divided into four subsections. The first subsection introduces the probabilistic model (Dynamic Bayesian Network [62]) we used for the inference, which describes the conditional dependencies between the relevant stochastic variables (e.g., states, actions, and subgoals). Importantly, at difference with most planning-as-inference architectures, our model includes (a probability distribution over) subgoals. The second subsection thus illustrates two methods that we used to define the “priors” of such subgoal states. The former (“algorithmic”) method, which is based on calculability theory, constitutes a novelty of our approach. In the experimental section of this article, we use it to explain human performance in the ToH. The latter (“perceptual”) method incorporates a simpler, distance-based metric of the ToH problem space. In the experimental section of this article, we use it to explain failures in human problem solving (e.g., problems in executing counterintuitive movements).

The last two subsections explain in detail the two nested procedures that compose the probabilistic inference: an “inner” procedure that produces candidate plans (see Algorithm 1) and an “outer” procedure that implements a decision rule to select among them (see Algorithm 2). Essentially, in the “inner” procedure, several candidate plans are stochastically produced by our subgoaling-based probabilistic method and scored according to informational measures. In the “outer” procedure, a Bayesian voting procedure selects the best-posterior-valued candidate plan [63, 64].

### Probabilistic model

**Fig 2:**
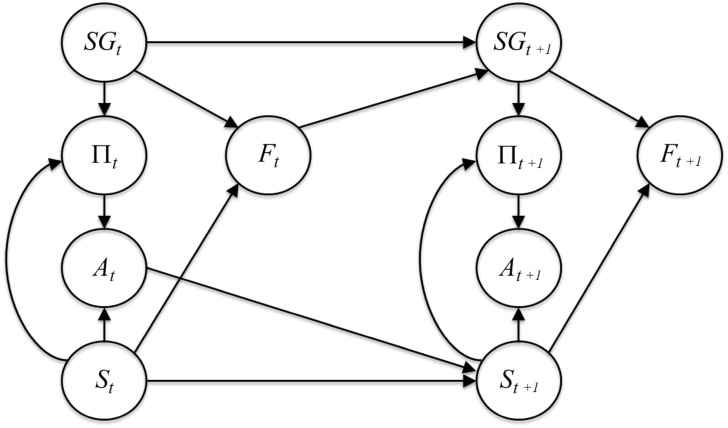
Graphical model used for the simulations. The graphical model is a Dynamic Bayesian Network [62] and expresses the probabilistic relations between variables. See the main text for explanation.

The probabilistic model used in the simulations is a Dynamic Bayesian Network (DBN) of Fig 2. The nodes of the DBN are arranged on two layers corresponding to two consecutive slices of time indicated with subscripts, e.g. *S*_*t*_ and *S*_*t*+1_. First-order and stationary Markov properties hold: every variable depends exclusively on other variables expressed at the same or in the immediately preceding time step.

The DBN model permits to formulate a problem solving task as a *Markov Decision Process* (MDP), in which states and actions are described by the stochastic variables *S* and *A*, respectively.

We assume that *S* varies in a discrete set consisting of integer values in the range {0, …, *n*}, with *n* being the total number of states. The node *A* corresponds to seven different actions {*act*1 − *act*6, *ε*}: move a disk from the first rod to the second rod (*act*1); move a disk from the first rod to the third rod (*act*2); move a disk from the second rod to the first rod (*act*3); move a disk from the second rod to the third rod (*act*4); move a disk from the third rod to the first rod (*act*5); move a disk from the third rod to the second rod (*act*6); and an auxiliary “rest” action (*ε*), i.e, a transition from a state to the same state. Note that not all actions are defined in every state (e.g., in some states it is impossible to move a disk from the first rod to the second rod).

The node Π in Fig 2 represents *policies*, or deterministic mappings from states to actions to be taken in these states. As a policy deterministically specifies an action for every state (*a* = *π*(*s*)), executing the same policy for a number of steps determines a sequence of transitions amongst states [61]. The total number *m* of policies available depends on the number of states *S* and actions *A* in the environment. We include also a *rest policy* *π*_*ε*_ that associates the action *ε* to every state (i.e., *ε* = *π*(*s*)).

Policy selection is modeled by a transition probability distribution *p*(Π|*s*, *sg*), where *s* is one arbitrary state and *sg* represents one arbitrary *subgoal*. Potentially, every state *s* could be a subgoal and used for planning a strategy in order to achieve the final goal. For this reason, in our simulations the set of subgoals has the same cardinality as the set of states: {0, …, *n*}. The final goal state is considered as a particular subgoal having the highest a priori probability.

At each time step, a new state is determined based on the current state and the action given by the selected policy, according to *p*(*S*_*t*+1_|*s*_*t*_, *a*_*t*_). The subgoal transition follows instead the distribution *p*(*SG*_*t*+1_|*f*_*t*_, *sg*_*t*_). The variable *F* monitors the agent’s *progress* in the task by reporting whether or not a goal or subgoal has been achieved, and determines when the inferential process terminates or a new subgoal needs to be sampled. *F* can only assume three discrete values: 0, 1, 2, see [38] for a related method. It has value 2 if the agent has reached the final goal state (in which case, the inferential process terminates). It has value 1 if the agent has just reached a subgoal state (in which case, a new subgoal is sampled). Otherwise, *F* has value 0 (in which case, the same subgoal is used for the next inferential step).

### Subgoal *a priori* distribution

Subgoals are states that are inferred during the *planning-as-inference (PAI)* process, and which enable the selection of optimal sequences of transitions from the initial state to the final goal.

A key feature of our probabilistic model is the use of a *subgoal a-priori probability distribution (SG)* that is used to guide the inferential process and in particular to select candidate subgoals (see later). For each state, this distribution essentially encodes a (prior) probability that the state is a potentially useful subgoal, or in other words a good way to carve the search problem.

In the following, we show two ways to calculate the a-priori probability (*prior*) of subgoal states. The former method (Algorithmic priors), crucial in our approach, is deduced from Algorithmic Probability theory, introduced by Solomonoff [65, 66]; as we will discuss, it reveals structural aspects of the problem space and affords efficient path planning. The latter method (Perceptual priors), carves the problem space in a different (and suboptimal) way, essentially encoding the mere perceptual distance to the goal state. We will use this latter method to simulate human failures in the execution of counterintuitive movements (see the “Results” section).

#### Algorithmic priors

The “algorithmic priors” of a state encode the idea that a state is a potentially useful subgoal if it affords several and efficient paths to goals—which, using algorithmic probability theory, can be defined in terms of the notion of *programs*, which we formally introduce next.

If we set a starting state *s*_*i*_, an arrival state *s*_*k*_ and a policy *π*_*j*_ (i.e., a function *π*(*s*) = *a* with *s* ∈ *S* and *a* ∈ *A*), the assumption of a discrete state space let us compute a list of actions that starting from *s*_*i*_ bring to *s*_*k*_ through a path *w* = 〈*s*_*i*_, …, *s*_*k*_〉. In other words, the object individuated by means of the triple (*s*_*i*_, *π*_*j*_, *s*_*k*_) is a *program* (i.e., a code that with *s*_*i*_ as input stops in *s*_*k*_). Notice that this is not a biunique correspondence because, if one fixes a path *w*, the program generating *w* can be determined by different policies (say, for instance, two policies *π* and *π*′ such that *π*(*s*) = *π*′(*s*) for every *s* ∈ *w* and $\exists \;\bar{s}\;\notin \;w:\pi \left(\bar{s}\right)\neq \pi^{\prime }\left(\bar{s}\right)$).

To understand the relation between programs and policies, let’s consider that, given a domain with transitions determined by a function *f* such that *f*(*s*, *a*) = *s*′, and once two states *s*_*i*_ and *s*_*k*_ are fixed, it is possible to generate a sequence of instructions to move from *s*_*i*_ to *s*_*k*_ via *π* by means of the following recursive definition:

$$\begin{array}{c} \begin{array}{c} s^{\left(0\right)}=s_{i}\\ s^{\left(\bar{t}\right)}=s_{k}\\ \pi \left(s^{\left(t\right)}\right)=a^{\left(t\right)}\\ f\left(s^{\left(t\right)},a^{\left(t\right)}\right)=s^{\left(t+1\right)} \end{array} \end{array}$$

The resulting instruction sequence can be represented as an *imperative program* for a register machine with *s*_*i*_ and *s*_*k*_, as input and output registers, respectively, and the other states *s*^(*t*)^ as destination/operand registers. As we discuss next, the length of a program can be converted into a probability and used within our probabilistic inference scheme.

Following principles of Algorithmic Probability theory, each program (*s*_*i*_, *π*_*j*_, *s*_*k*_) can be formally transformed into a *code* (i.e., a binary string p_*s*_*k*__(*s*_*i*_, *π*_*j*_) of length |p_*s*_*k*__(*s*_*i*_, *π*_*j*_)|) and processed by a computing machine. To each code p_*s*_*k*__(*s*_*i*_, *π*_*j*_), and consequently to each program, it is possible to assign a probability 2^−|p_*s*_*k*__(*s*_*i*_, *π*_*j*_)|^ depending on its length. In other words, the probability 2^−|p_*s*_*k*__(*s*_*i*_, *π*_*j*_)|^ is the probability of a program code that starts from *s*_*i*_ and ends up in *s*_*k*_, after executing the sequence of actions *a*_1_, …, *a*_*m*_ that is dictated by the policy *π*_*j*_.

This probability goes to 0 in the limit of |p_*s*_*k*__(*s*_*i*_, *π*_*j*_)| → ∞, when a program does not halt in *s*_*k*_ (or it does not halt at all). Furthermore, this probability is equal to 1 when |p_*s*_*k*__(*s*_*i*_, *π*_*j*_)| = 0 (this happens when initial and arrival states coincide). This last condition is satisfied in our representation if and only if there exists a policy working as an “identity policy” corresponding to our definition of the policy *π*_*ε*_, see the Section on “Probabilistic Model” for details.

The algorithmic probability for a generic state *s*_*k*_ can be computed, up to a normalization factor, by the following Equation:

$$P\left(SG=s_{k}\right)\propto \sum_{i}\sum_{j}2^{-|p_{s_{k}}\left(s_{i},\pi_{j}\right)|}$$ (1)

Eq (1) applied on every state generates an a priori “algorithmic probability” distribution (*algorithmic priors*) in which the more informative states (i.e., subgoals important for decomposing any multi-step planning task) have the highest probability values. This method is related to the approach presented in [43], which considers for each state “the amount of Shannon information that the agent needs to maintain about the current goal at a given state to select the appropriate action”.

The computed algorithmic priors for subgoal states are specific for a given environment and their computation is very costly. In [35] we have empirically verified that sampling on a much smaller set of policies (100.000 in [35]) is sufficient to have a consistent approximation of the prior values and to reveal the shape of the distribution (i.e., which states have higher prior values). Sampling increasingly more policies allows to straightly improve this approximation thanks to the “cumulative” nature of Eq (1). Either way, we designed an efficient strategy for their non-approximated evaluation. First, algorithmic priors can be computed in an off-line (batch) mode, before inference, and reused for every inference in the same environment. Second, we use joint probabilities and an adequate state space representation to make the computation of algorithmic priors more effective. In fact, Eq (1) can be formulated in terms of algorithmic joint probabilities as

$$P\left(SG=s_{k}\right)\propto \sum_{i}p\left(s_{k},s_{i}\right)$$ (2)

with

$$p\left(s_{k},s_{i}\right)\propto \sum_{l}\sum_{j}2^{-(|p_{s_{k}}\left(s_{l},\pi_{j}\right)|+|p_{s_{i}}\left(s_{l},\pi_{j}\right)|)}=\sum_{j}2^{-|p_{s_{k}}\left(s_{i},\pi_{j}\right)|}.$$ (3)

where the equality on the right side follows by two properties of the model: 1) two states co-occur if and only if the programs that have *s*_*k*_ (*s*_*i*_) as output are prefixes of the programs returning *s*_*i*_ (*s*_*k*_); and 2) if a program from *s*_*k*_ to *s*_*i*_ exists then, by assuming that transitions are symmetric, there is also the opposite one from *s*_*i*_ to *s*_*k*_.

Additionally, the space of states can be rearranged as a graph with the states *s*_*i*_ as vertices and the transitions between states as edges. Encoding the graph as an *adjacency list*, i.e., a collection of unordered lists related to each state and composed of the neighbors of the related vertex, it is possible, given two states *s*_*i*_ and *s*_*k*_, to run a standard depth-first search algorithm for finding all the paths *c* ∈ *C*(*s*_*i*_, *s*_*k*_) between them. Every path *c* corresponds to a distinct program code p_*c*_, but, as we stressed previously, each program can be attained by a certain set of different policies indicated as *μ*(p_*c*_). The number *μ*(p_*c*_) can be computed by considering each combination of the neighbors of the states not involved in the specific path *c*.

Consequently, the joint probability *p*(*s*_*k*_, *s*_*i*_) expressed in Eq (3) can be written in the following form:

$$p\left(s_{k},s_{i}\right)=\sum_{c\in C(s_{k},s_{i})}\mu \left(p_{c}\right)\left(2^{-|p_{c}|}\right).$$ (4)

In our case the number of states is finite, thus, given any pair of states, it is possible to compute their joint probability using Eq (4). By substituting Eq (4) in Eq (2) we obtain:

$$P\left(SG=s_{k}\right)\propto \sum_{i}\sum_{c\in C(s_{k},s_{i})}\mu \left(p_{c}\right)\left(2^{-|p_{c}|}\right)$$ (5)

This Eq (5) allows computing for each state *s*_*k*_ the value *p*(*SG* = *s*_*k*_) corresponding to the algorithmic prior assigned to the state *s*_*k*_ and expressed in Eq (1). Note that Eq (5) dramatically reduces the computational complexity of Eq (1) to *O*(*n* + *e*), equal to the depth-first search complexity.

The same procedure based as an adjacency list is employed in the inferential process described below (see the Section on “Inferential procedure”) to calculate *p*(*sg*_*t*+1_ | *sg*_*t*_) and *p*(*s*_*goal*_ | *sg*_*t*+1_) through the usual chain rule for probabilities.

#### Perceptual priors

In human problem solving, a perceptual strategy is one in which subjects privilege the moves whose effect more closely (perceptually) resembles the goal state: the larger is the number of disks on the correct rod, the higher is the probability assigned to the corresponding state. In keeping, and similar to previous modeling work [67], we formalized a “perceptual metric” that favors disks put on the target rod. To this aim, we encoded states of the problem as vector **v**(*s*) = (*rod*_1_, …, *rod*_*d*_, …, *rod*_*D*_), where *rod*_*d*_ denotes the number of the rod on which the disk is, and *d* denotes the index of the disk type (e.g., in our tests *D* = 3 and S27 and S20 are mapped into (3, 3, 3) and (2, 2, 2), respectively). The perceptual prior of the state *s*_*i*_ can thus be given as:

$$p_{\text{perc}}\left(s_{i}|s_{\text{goal}}\right)=\frac{\exp(-||v\left(s_{i}\right)-v\left(s_{goal}\right)|{|}_{1})}{\sum_{l}\exp(-||v\left(s_{l}\right)-v\left(s_{goal}\right){\left|\right|}_{1})}$$ (6)

where we use the $L_{1}$ norm between vectors to calculate the distance between *s*_*i*_ and *s*_*goal*_.

### Inferential procedure

In the standard *planning-as-inference (PAI)* approach, the inference tries to find a suitable policy from a start (*s*_0_) to a goal location (*s*_*goal*_). Rather, in keeping with the recognized importance of subgoaling in problem solving, our method infers a series of simpler sub-plans that pass through a sequence of subgoals *s*_0_, *sg*_1_, …, *sg*_*k*_, …, *s*_*goal*_, until the goal state is reached. In other words, here the inferential process aims at finding a sequence of subgoals (and associated policies that govern the transitions from one subgoal to the next) that best permits to solve a specific problem, rather than finding a solution from start to end. The way the sequence of subgoals and associated policies are selected is described next.

The procedure iteratively *samples* (i.e., extracts probabilistically), first, candidate subgoals from the previously described a priori subgoal distribution *p*(*SG*), and second, policies (from *π*) that can govern the transition to the sampled subgoal. The sampling procedure is cycled until the final goal *s*_*goal*_ is eventually reached (or for a maximum number of steps *T*_max_). During this procedure, both *s* and *p*(*SG*) are iteratively updated to reflect the current planned path toward the goal and the most useful subgoals given the final goal, respectively.

In the course of this iterative process, the candidate subgoals are retained or discarded by considering the computational complexity of the sub-problems they generate. More formally, a candidate subgoal sequence is selected on the basis of the code length of the corresponding *programs*, which go from one subgoal (or the start location) to the next. We remind that a *program* is defined as the sequence of actions necessary for the transition from an initial state *s* to a subgoal state *sg* (and is equivalent to a path following a policy *π* [61] from *s* to *sg*). The length of the program (i.e., the number of actions necessary to reach *sg* from *s*) is converted into a probability using *algorithmic probability theory* [65, 66, 68, 69] and this probability is used in the iterative procedure to decide whether the solution (including the subgoal plus the program) should be retained or discarded.

The inference is formalized by the pseudocode of Algorithm 1. Following the model of Fig 2, the inference starts at time *t* = 0 from the initial state *s*_0_ of the node *S*. The distribution of the algorithmic priors *p*(*SG*) is modified to set the goal state *s*_goal_ as the state with highest prior.

**Algorithm 1** Planning Inference(*s*_0_, *s*_goal_, *q*, *p*(*SG*), *T*_max_)

**Require:** Starting state *s*_0_, goal state *s*_goal_, sampled model instances *q*, subgoal algorithmic priors *p*(*SG*), maximum number of forward inferences *T*_max_.

**Ensure:** State sequence [*s*_0_, …, *s*_goal_], subgoal sequence *Seq*.

1: *t* = 0

2: **set***S*_0_ to the starting state *s*_0_

3: **sample** a subgoal *sg*_0_ from the prior distribution *p*(*SG*) attained by using Eq (1) on each state

4: **select** a policy *π*_0_ maximizing Eq (8) sampled through a Monte Carlo method

5: **determine** the action *a*_0_ depending on *π*_0_ and *s*_0_

6: **evaluate** the termination condition state *F*_0_ according to *p*(*F*_0_|*sg*_0_, *s*_0_)

7: **while** (*F*_*t*_ < 2 and *t* ≤ *T*_max_) **do**

8:  *t* = *t*+1

9:  **determine** the state *s*_*t*_ by means of *p*(*S*_*t*_|*a*_(*t* − 1)_, *s*_(*t* − 1)_)

10:  **select** the subgoal *sg*_*t*_ maximizing Eq (12) sampled through a Monte Carlo method

11:  **select** a policy *π*_*t*_ maximizing Eq (8) sampled through a Monte Carlo method

12:  **determine** the action *a*_*t*_ depending on *π*_*t*_ and *s*_*t*_

13:  **evaluate** the termination condition variable *F*_*t*_ according to *p*(*F*_*t*_|*sg*_*t*_, *s*_*t*_)

14:  **update** subgoal prior distribution by posing *p*(*SG*_*t*_ = *s*_*t*_) = 0

15: **end while**

The distribution *p*(*SG*) is initially determined by using Eq (1) to assign an algorithmic prior value to each state of the environment. Therefore, we sort in descending order the values of *p*(*SG*) and impose that

$$P\left(SG=s_{goal}\right)\propto \max\left(p\left(SG\right)\right)+\Delta$$ (7)

where Δ > 0 is the maximum difference, in absolute value, between two consecutive priors arranged by the ordering.

Note that the maximization of Eq (7) operates over the values of *p*(*SG*) calculated using Eq (1), which are predefined during the inference. Afterwards, the whole prior distribution is normalized taking into account the modified value for *P*(*SG* = *s*_*goal*_).

At time *t* = 0, a subgoal *sg*_0_ is drawn from the a priori algorithmic probability distribution (line 3) presented in the Section on “Algorithmic priors” and modified as in Eq (7). Then, the instruction in line 4 searches for a policy *π*_*t*_ such that it is possible to build a program representing a transition from *s*_*t*_ to *sg*_*t*_. This is achieved by drawing a policy from the following probability distribution:

$$p\left(\Pi_{t}=\pi_{j}\;|\;s_{t},sg_{t}\right)\propto p\left(sg_{t},s_{t}\;|\;\Pi_{t}=\pi_{j}\right)\;p\left(\pi_{j}\right).$$ (8)

Eq (8) expresses the probability of selecting a specific policy *π*_*j*_ in function of the length of the *program code* that from the current state *s*_*t*_, by means of *π*_*j*_, brings to the subgoal *sg*_*t*_, weighted with the prior of the policy *π*_*j*_

It is possible to rewrite Eq (8) in an algorithmic form as:

$$\begin{array}{ccc} p(\Pi_{t}=\pi_{j}|s_{t},sg_{t}) & \propto & p\left(sg_{t},s_{t}\;|\;\Pi_{t}=\pi_{j}\right)\;p\left(\pi_{j}\right)\\ & \propto & \left(2^{-|p_{sg_{t}}\left(s_{t},\pi_{j}\right)|}\right)p\left(\pi_{j}\right) \end{array}$$ (9)

where the first factor of the right side product derives from the algorithmic joint probability shown in Eq (3) by considering the policy *π*_*j*_ as fixed. Hence, in the resulting Eq (9), the probability of a specific policy *π*_*j*_ is proportional to its capability to generate a program that starts from the current state *s*_*t*_ and reaches the currently selected subgoal *sg*_*t*_.

Note that Eq (9) is symmetric in the route as the transitions are symmetric—but the fact that we assign the final goal a high prior gives inference a directionality.

According to Eq (9), the probability of a policy *π* conditioned by a state *s* and a subgoal *sg* is the product between the likelihood that *π* generates a path from *s* to *sg* and the a priori probability *p*(*π*). By assuming that *p*(*π*) is uniform, for any given pair of *s* and *sg*, there exists a subset of policies that have the same probability.

In our simulations, drawing from the distribution defined in Eq (9) takes place by means of a Monte Carlo sampling method [70]. The candidate policies are sampled from the uniform distribution *p*(Π_*t*_) and then the policy corresponding to π* = arg max_*j*_ *p*(*π*_*j*_|*s*_*t*_, *sg*_*t*_) is selected. This is the best policy (i.e., the one resulting in shortest paths) amongst those that were sampled. Of note, various alternatives to sampling methods (e.g., heuristic techniques or tree search [71]) can be adopted.

The inference described so far starts from an initial (clamped) state *s*_*t*_ and returns a subgoal *sg*_*t*_ and a policy *π*_*t*_ able to reach it. (This can be viewed as an Option-like plan built on-the-fly with the minimum number of actions involved into the transition from the initial to the subgoal state.) Given the policy *π*_*t*_, and knowing the state *s*_*t*_, the action *a*_*t*_ is determined in a straightforward manner (line 5).

At this point—line 6—the node *F*_*t*_ checks the reaching of a goal state *s*_*goal*_. If *s*_*t*_ = *s*_*goal*_ then *f*_*t*_ = 2 and inference process stops, otherwise it proceeds until at least one among the termination criteria is fulfilled (line 7): either the node *F*_*t*_ evaluates to 2 or a maximum number *T*_*max*_ of inferential cycles has been effected.

During these (from line 7 to line 15) and after that the next state *s*_*t*+1_ is determined via *p*(*s*_*t*+1_|*s*_*t*_, *a*_*t*_) Table 1 (line 9), the subgoal transition *SG*_*t*_ → *SG*_*t*+1_[38] is established (line 10). In case that *s*_*t*_ ≠ *sg*_*t*_, the node *F*_*t*_ assumes a zero value and the subgoal *sg*_*t*+1_ is forced to be the same as time *t*. Contrarily, when *f*_*t*_ = 1, the current state *s*_*t*_ is equal to the current subgoal and a new one must be found.

**Table 1:** Algorithmic parameters set in the experiment shown in Fig 4.

Experiment	Tower of Hanoi Task
States	27
Actions	7
Policies	∼2.3 ⋅ 10^12^
Particles	100
*R*	10
*Θ*	0.8
*T*_*max*_	12
*G*_*res*_	30%

In order to guide subgoal determination towards the goal state *s*_*goal*_, the inference “clamps” the current subgoal *sg*_*t*_, and assumes that *f*_*t*+1_ = 2, namely it fictively considers the goal state as observed. Therefore, by the aforementioned considerations, the distribution *p*(*SG*_*t*+1_ | *f*_*t*+1_ = 2, *sg*_*t*_) can be stated as:

$$\begin{array}{ccc} p(sg_{t+1}\;|\;f_{t+1}=2,sg_{t}) & \propto & p\left(f_{t+1}=2\;|\;sg_{t+1}\right)\;p\left(sg_{t+1}\;|\;sg_{t}\right)\\ & \equiv & p\left(s_{goal}\;|\;sg_{t+1}\right)\;p\left(sg_{t+1}\;|\;sg_{t}\right) \end{array}$$ (10)

The term *p*(*sg*_*t*+1_ | *sg*_*t*_) estimates the probability that the subgoal *sg*_*t*+1_ is chosen after *sg*_*t*_ and the likelihood *p*(*f*_*t*+1_ = 2 | *sg*_*t*+1_) corresponds to the conditional probability *p*(*s*_*goal*_ | *sg*_*t*+1_) of the goal state *s*_*goal*_ with respect to the subgoal *sg*_*t*+1_.

The algorithmic expression of Eq (10) stems from conditioning all the programs returning the goal to produce *sg*_*t*+1_ as intermediate outcome; thus, it becomes:

$$\begin{array}{ccc} p(sg_{t+1}\;|\;f_{t+1}=2,sg_{t}) & \propto & p\left(s_{goal}\;|\;sg_{t+1}\right)\;p\left(sg_{t+1}\;|\;sg_{t}\right)\\ & \propto & \frac{1}{P\left(sg_{t+1}\right)P\left(sg_{t}\right)}\left(\sum_{j}2^{-|p_{s_{goal}}\left(sg_{t+1},\pi_{j}\right)|}\right)\left(\sum_{j}2^{-|p_{sg_{t+1}}\left(sg_{t},\pi_{j}\right)|}\right) \end{array}$$ (11)

Candidate subgoals *sg*_*t*+1_ are sampled by a Monte Carlo process [70] from the subgoal prior distribution *p*(*SG*_*t*_). The subgoal at time *t* + 1 is calculated as sg* = arg max_*k*_ *p*(*sg*_*k*_ | *f*_*t*+1_ = 2, *sg*_*t*_).

Summing up, the *SG*_*t*+1_ subgoal state is determined by means of a posterior estimation in dependence on the values of *s*_*t*_ and *sg*_*t*_, according to the equation:

$$p\left(SG_{t+1}|f_{t},sg_{t}\right)=\left\{\begin{array}{cc} \delta_{sg_{t+1},\;sg_{t}} & \text{if }f_{t}=0\\ p(SG_{t+1}\;|\;f_{t+1}=2,sg_{t}) & \text{if }f_{t}=1\\ \delta_{sg_{t+1},\;s_{goal}} & \text{if }f_{t}=2 \end{array}\right.$$ (12)

where *δ*_*a*, *b*_ is 1 when *a* = *b* while is 0 otherwise.

Once the state for *SG*_*t*+1_ has been set, the inference proceeds from line 11 to line 13 by, according to this sequence and following the methods previously discussed, selecting a policy *π*_*t*+1_, an action *a*_*t*+1_, and assessing *f*_*t*+1_.

Finally, in the instruction at line 14, subgoal prior distribution is updated by ‘switching off’ the prior of the current state, i.e., setting the probability *p*(*SG*_*t*_ = *s*_*t*_) to zero and normalizing the whole distribution. This update has the effect of an ‘on-line memory’ preventing the inference from selecting at the line 10 previously visited states as subgoals.

In sum, the output of the iterative sampling procedure described in Algorithm 1 is a planning sequence *Seq*. Importantly, this procedure is conducted in parallel by multiple *particles* of a particle-filtering algorithm (with resampling) [70]. Each particle runs through all the problem space (for a maximum number of steps *T*_max_) and returns a sequence of subgoals and associated programs that solve the problem (for the sake of simplicity, this ensemble of particles continues inferring paths until a percent *G*_res_ of them achieves the goal). Note that although the subgoaling procedure usually splits the problem into smaller sub-problems, it is also possible that a given particle finds a solution that does not require subgoals.

In the next subsection we describe how the to-be-executed plan is selected based on a mechanism that accumulates the “votes” of all the particles that solve the problem.

### Decision making through posterior accumulation scoring

The inferential procedure illustrated in Algorithm 1 permits to probabilistically solve planning problems. Nevertheless, its nature is essentially stochastic because a sampling technique is used to draw both policies and subgoals. Consequently, given the same pair of goal and start states, the model will infer more than one possible path as a response. This creates a problem of *plan selection*, because ultimately the agent can only select and execute one single plan.

We model this selection problem in terms of an accumulation-to-bound process, in which at every iteration each particle reaching the goal brings a “vote” for its candidate plan. Specifically, we use a variant of the widely adopted drift-diffusion model [72] that casts choice as a Bayesian sequential analysis over multiple alternatives [63, 64, 73]. In this approach, a set of different planning hypotheses is sequentially analyzed by gathering evidence for each of them during their execution. The sequential analysis is stopped when the results become significant according to a pre-defined termination criterion. The posteriors for the planning hypotheses are updated by using Bayes’ rule until reaching a threshold value.

This method is implemented via a *particle filtering* algorithm with *resampling* reported in Algorithm 2 (see [70]). At each resampling step *r*, for a maximum number of *R*, a set *Q*_*r*_ = {*q*^(1)^, …, *q*^(*K*)^} of particles representing *K* distinct planning hypotheses of the internal model activation is generated (line 7) and tracked forward until a time *T*_*R*_. By applying Algorithm 1 at the line 10, a subset of *H* ≤ *K* particles, able to successfully infer a plan directed to the goal, is carried out and the related subgoal sequences *Seq*_*h*_ = [*sg*_*t* = 0_, …, *sg*_*t* = *T*_*r*__]_*h*_, for *h* = 1, …, *H*, are evaluated by means of a score *θ*_*h*_.

**Algorithm 2** Sequential Decision Making(*s*_0_, *s*_goal_, *K*, *T*_max_, Θ, *R*, *G*_res_)

**Require:** Starting state *s*_0_, goal state *s*_goal_, particle number *K*, maximum number of forward inferences *T*_max_, decision threshold Θ, number of resamplings *R*, particles-gone-to-goal threshold *G*_res_.

**Ensure:** Subgoal sequence probability distribution.

1: *r* = 0

2: **initialize** the set of subgoal sequences Σ ≡ {*Seq*_*h*_}_*h* = 1, …, *H* ≤ *K*_ = ∅

3: **initialize***θ*_*h*_(0)

4: **compute***p*_0_(*SG*) by means of Eq (1) and Eq (7)

5: **while** (*θ*_*h*_(*r*) ≤ Θ, ∀*h* and *r* < *R*) **do**

6:  *k* = 0

7:  **create** the sample set *Q*_*r*_ = {*q*^(1)^, …, *q*^(*K*)^}

8:  **while** (*k* ≤ *K* and *G*(*Q_r_*)<*G*_res_) **do**

9:   *k* = *k*+1

10:   *Seq*_*h*_ = Planning Inference(*s*_0_, *s*_goal_, *q*^(*k*)^, *p*_r_(*SG*), *T*_max_)

11:   Σ = Σ ∪ *Seq*_*h*_

12:  **end while**

13:  *r* = *r* + 1

14:  **update** votes *θ*_*h*_(*r*) for the sequences in Σ set by Eq (13)

15:  **update** subgoal prior distribution *p*_*r*_(*SG*) by Eq (14)

16: **end while**

Initially, when *r* = 0, we assume that *p*_*r*_(*SG*) is computed through Eq (1) and Eq (7) and that, additionally, *θ*_*h*_(0) = *p*(*Seq*_*h*_) where *p*(*Seq*_*h*_) is the prior distribution on the potential subgoal sequences. It is possible to assign a prior distribution for the sequences on the basis of the information extracted from the specific state space; on the other hand, this prior can be flattened when no additional information is present.

This inferential process is executed until at least one of the *inference terminating conditions* is reached (from line 8 to line 12): either *G*(*Q*_*r*_) (the percent of particles in *Q*_*r*_ reaching the goal) is greater than or equal to a given threshold *G*_res_, or the inference reaches a (fixed) maximum number *T*_max_ of iterations.

Subsequently, the scores of the subgoal sequences *Seq*_*h*_ are updated at the line 14 using the recursive formula:

$$\theta_{h}\left(r+1\right)\propto \theta_{h}\left(r\right)\cdot p\left(Q_{r}|Seq_{h}\right)$$ (13)

where

$$p\left(Q_{r}|Seq_{h}\right)=\frac{1}{K}\sum_{k}^{K}p\left(q^{\left(k\right)}|Seq_{h}\right)$$

is the *evidence* of the current particle set *Q*_*r*_ given the sequence *Seq*_*h*_, computed as the proportion of particles *q*^*k*^ tracing the specific subgoal sequence *Seq*_*h*_. Therefore, the scores *θ*_*h*_ have the meaning of posteriors on *Seq*_*h*_ accumulated in *r* steps.

Each step *r* concludes by updating, in line 15, the subgoal priors *p*_*r*_(*SG*) on the basis of the sampled sequences:

$$p_{r+1}\left(SG=sg_{k}\right)\propto p\left(Q_{r}|sg_{k}\right)\cdot p_{r}\left(SG=sg_{k}\right)$$ (14)

where *p*(*Q*_*r*_|*sg*_*k*_) is the rate of the particles numbering *sg*_*k*_ among the subgoals exploited.

At the successive step *r* + 1, a new set of hypotheses *Q*_*r*+1_ is resampled by adopting *p*_*r*+1_(*SG*) as subgoal prior distribution, which increases the probability of selecting the more effective subgoal sequences at the next step. This procedure is iterated until one of the convergence criteria is met (line 5): (a) a subgoal sequence receives a total score greater than or equal to a predefined *decision threshold* Θ, or (b) the maximum number of iterations *R* is performed. In both cases, the sequence with the highest score is selected for execution.

To verify if the proposed computational model can successfully reproduce human problem solving strategies, we tested it in three representative ToH tasks that are widely used to study how humans solve (or fail to solve) structured problems.

## Results

### Humans are sensitive to the community structure of the ToH

A first important finding that we address here is the fact that subjects that solve a ToH have been found to be very sensitive to the *community structure* of the problem [74]. As shown in Fig 1C, a ToH has a community structure composed of three clusters of nodes separated by so-called “bottlenecks”, viz. states *S*9 − *S*11, *S*6 − *S*10, and *S*23 − *S*24. The bottlenecks are here defined topologically as narrow segments bridging between densely interconnected clusters of vertices; in other words, bottlenecks are the only way to pass from one cluster to another. For example, the bottleneck *S*9 − *S*11 is the only way to pass from the top cluster to the bottom-left cluster or vice versa.

Previous research has identified the importance of community structures in carving problem spaces [75] and the ToH is no exception. In a series of empirical studies on human problem solving [74], participants were asked to solve a ToH problem equivalent to navigating from *S*11 (starting state) to *S*13 (goal state), see Fig 1C. It is possible to note that there are two shortest-path solutions to the problem that require the same (minimal) number of steps: the former is *w*_1_ = 〈*S*11, *S*14, *S*18, *S*24, *S*23, *S*17, *S*13〉 and the latter is *w*_2_ = 〈*S*11, *S*9, *S*8, *S*7, *S*6, *S*10, *S*13〉. If the number of steps were the only determinant of behavior, participants should select the two paths with the same probability. However, there is an important difference between the two paths: the former requires traversing one bottleneck (i.e., one boundary between two communities, *S*23 − *S*24) while the latter requires traversing two bottlenecks (*S*9 − *S*11 and *S*6 − *S*10). Thus, if participants take the community structure into consideration when they plan and prefer remaining in the same cluster of nodes (i.e., not traversing bottlenecks) when possible, they should prefer the former solution (*w*_1_) with higher probability. Participants selected *w*_1_ in 72% of the cases, suggesting that they are sensitive to the community structure of the ToH and prefer remaining in the same community.

The fact that there are two shortest-path solutions but one is consistently preferred indicates that the probability to select a path does not simply depend on its “physical” length (i.e., the number of steps). Instead, it might be also sensitive to the informational / community structure of the environment (e.g., the transition probabilities or the presence of community boundaries). To put it in another way, in problem solving, a path between two points (start and end of the problem) is not necessarily represented (or calculated) in a metrical space that only encodes their physical distance. Instead, it might be represented in a subtler “problem space” in which—say—the length of a path or the probability to select it depends on additional factors such as informational constraints, the cost of the inferential process required to solve the problem (e.g., find the path) or the amount of information required to encode or recall it [43–45, 74, 75].

Our proposed model incorporates a new hypothesis on how structural / informational constraints are incorporated in the inferential process. In our probabilistic approach, the prior subgoal probability shown in Fig 1C permits to identify bottlenecks or boundaries between communities in terms of paths that have low probability to be traversed. As the subgoal (prior) probability of the bottleneck nodes is smaller than the other nodes, any path traversing bottleneck nodes will have lower probability in our inferential system, discouraging subjects from crossing boundaries or traversing clusters of nodes. This stems from a key aspect of the algorithm: states having lower prior probability are more rarely sampled as subgoals during the subgoal selection phase (line 3 of Algorithm 1). Accordingly, the inferential system tends to select paths that have higher probability (or equivalently, correspond to shorter programs), which in turn are preferentially composed of high-probability states. Indeed, the probability of states enters into the evaluation of the policies that generate paths, see Eq 9. Note also that the prior probability distribution is graded, and the probability of nodes decreases in proximity to bottlenecks. This implies that not only traversing bottlenecks, but also going towards bottlenecks is less probable in our inferential system.

To illustrate the effects of this subgoal-induced community structure in quantitative terms, we compared our model with subjects’ performance as reported in [74]. Let’s consider again the problem of starting from *S*11 to reach the goal state *S*13 (see Fig 1C). Considering the two possible shortest-path solutions, (*w*_1_ = 〈*S*11, *S*14, *S*18, *S*24, *S*23, *S*17, *S*13〉 with only one community-traverse, and *w*_2_ = 〈*S*11, *S*9, *S*8, *S*7, *S*6, *S*10, *S*13〉 with two community-traverses), the first path is made up of states with higher subgoal prior probability.

**Fig 3:**
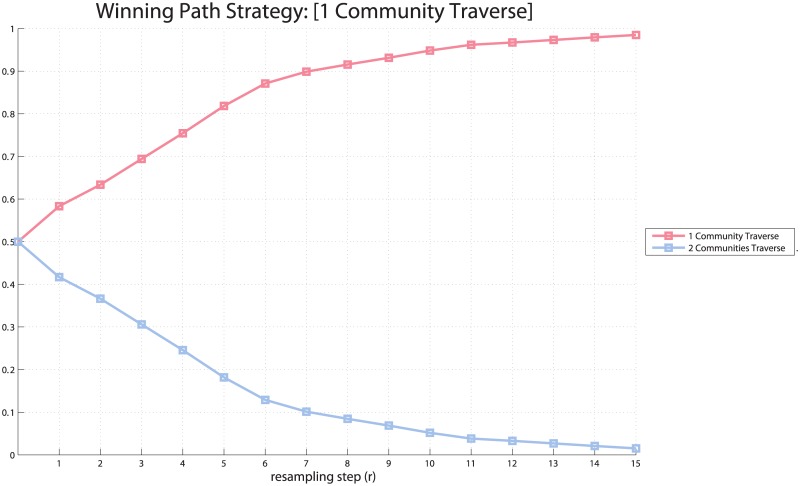
Decision making procedure. Choice between two paths (*w*_1_ vs. *w*_2_) from *S*11 to *S*13 that require the same number of steps but traverse a different number of bottlenecks (1 for *w*_1_ and 2 for *w*_2_. Choice is performed using a probabilistic version of the drift diffusion model [72] introduced in the Section on ‘Methods’, in which particles (of the particle filtering algorithm) that reach the goal “vote” for the specific strategy they followed.

Our simulations show that even in the presence of these two shortest-path solutions to this problem, each involving the same number of steps, the winning (i.e., most voted) strategy is *w*_1_: the one that selects the path traversing less bottlenecks or clusters. Fig 3 shows a dynamical competition between the two solutions in which particles “vote” for one of them (see Section on ‘Methods’). At each iteration (resampling) of the voting procedure the probability for *w*_1_ increases; note that it reaches the same proportion (72%) as reported empirically in [74] after four iteration steps. Of course, the exact fit of the data is not extremely important here, nor is the specific combination of start and goal states, as our findings generalize to any other problem in the ToH. What our results show is that the proposed method reproduces the subjects’ sensitivity to the community structure of the ToH by only appealing to probabilistic computations and a principled approach to establish which subgoals are potentially useful.

### Humans are sensitive to the *nested* structure of the ToH

The three-cluster structure described above is not the only community structure of the ToH. Rather, the ToH has a 3-level *nested* community structure with (triangular) sub-clusters, see Fig 1C. Nested within the aforementioned (level-3) clusters one can find three (level-2) clusters (e.g., in the top level-3 cluster, these correspond to {*S*1, *S*2, *S*3}, {*S*5, *S*8, *S*9}, and {*S*4, *S*6, *S*7}). Furthermore, nested within each level-2 cluster one can find three level-1 clusters that correspond to individual nodes (e.g., in the top level-2 cluster, the nodes *S*1, *S*2, and *S*3). Bottlenecks at different levels of depth correspond to these nested clusters: *S*2 − *S*3 is a level-1 bottleneck, *S*7 − *S*8 is a level-2 bottleneck, and *S*23 − *S*24 is a level-3 bottleneck.

It has been reported that this nested structure affects human behavior [74]. Specifically, the costs for traversing a level-3 bottleneck (*S*23 − *S*24) are higher than those for traversing a level-2 bottleneck (*S*7 − *S*8), which in turn are higher than those for traversing a level-1 bottleneck (*S*2 − *S*3)—where the costs are implicitly measured as longer reaction times required to make a decision in the ToH. Once again, this difference only exists in a “problem space” and not in the standard “metric space” that only measures the number of steps to reach a goal location, because traversing a bottleneck (independent of its level) only requires one step.

The sensitivity for the nested ToH structure can be explained within our framework if one considers that the prior probability of traversing *S*2 − *S*3 (*P*(*S*3|*S*2) ⋅ *P*(*S*2) = 0.0464) is higher than traversing *S*7 − *S*8 (*P*(*S*8|*S*7) ⋅ *P*(*S*7) = 0.0358), which in turn is higher than traversing *S*23 − *S*24 (*P*(*S*24|*S*23) ⋅ *P*(*S*23) = 0.0355), see Fig 1C. These results thus extend those reported in the former Section and illustrate how the structure of the prior subgoal distribution nicely captures key characteristics of the “problem space” that humans use to solve problems.

### Humans have difficulties to execute counterintuitive movements

Empirical studies of how humans solve the Tower of Hanoi have identified a specific deficit in the failure of executing *counterintuitive movements*: moves that are apparently in opposition to the end goal-state (e.g., remove a disk from the target rod) but that are necessary to achieve the goal efficiently. In these cases, two strategies have been identified that have opposite results: a “look-ahead” strategy that considers the long-run effects of the counterintuitive movements and their benefits for the overall problem solving, versus a “perceptual” strategy that only tries to decrease myopically the perceived (apparent) distance to the goal state (e.g., only increases the number of disks in the target rod) [57, 76]. This latter strategy disregards counterintuitive moves—which, by definition, require removing a disk from the target rod, thus apparently increasing the perceived distance from the goal state) and can lead to suboptimal behavior. Here we characterize both strategies from a common probabilistic inference viewpoint, and analyze why the former (look-ahead) permits efficient problem solving while the latter (perceptual) determines a failure of executing counterintuitive movements.

**Fig 4:**
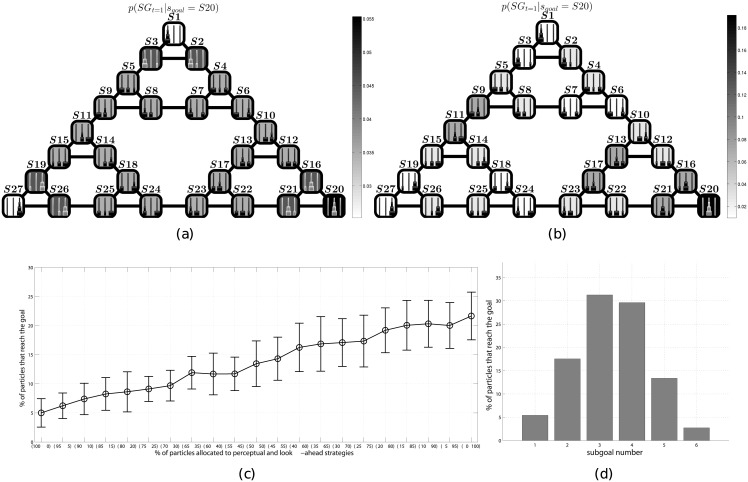
Simulation of look-ahead vs. perceptual strategies. (a) Probability distribution on *SG* given the goal *S*20 using the “look-ahead” strategy. The priors are calculated by starting from the priors shown in Fig 1C, assigning the goal state (*S*20) the highest probability (see Eq 7), and then normalizing. (b) Probability distribution *SG* given the goal *S*20 using the “perceptual” strategy. Different from the “look-ahead” strategy, here the priors reflect the “perceptual similarity” between each state and the goal state *S*20; for example, it is high for states in which there are disks in the second rod (e.g., *S*9, *S*11, *S*13, *S*17, *S*16, *S*21). As for the “look-ahead” strategy, it is highest in the goal state *S*20. The full procedure for calculating the priors is explained in the Section on Methods. (c) Probability of finding the best policy in function of the number of particles assigned to “perceptual” vs “look-ahead” strategy. The left part of the figure shows a “pure perceptual” strategy (100% of the particles use the priors of the “perceptual” strategy); the right part of the figure shows a “pure look-ahead” strategy (100% of the particles use the priors of the “look-ahead” strategy); and the central part of the figure shows intermediate cases (e.g., 50% of the particles use the priors of the “perceptual” strategy and 50% of the particles use the priors of the “look-ahead” strategy). Results are the mean of 25 runs. (d) Number of subgoals used by the particles (of the particle filtering algorithm) to solve the ToH problem starting from *S*27 and ending in *S*20. As this number also includes the last (goal) state, the figure shows that the majority of particles solve the problem using 2 or 3 intermediate subgoals. In principle, every state of the ToH can be a subgoal; however, our further analysis shown in Fig 7 highlights that the most selected path is one that uses *S*22 and *S*21 as subgoal states.

Let’s consider a sample problem consisting in going from a start (*S*27) to a goal (*S*20) configuration, see Fig 1. In our implementation, the two aforementioned (“look-ahead” vs. “perceptual”) strategies only differ for the choice of prior distributions of subgoals *p*(*SG*). The priors for the “look-ahead” strategy are shown in Fig 4A); they are calculated using the same approach as used in the former two studies. The priors for the “perceptual” strategy are shown in Fig 4B. Here, in keeping with the problem solving literature, the probability of a movement is computed using a perceptual-based proximity criterion: the more disks the agent sees on the correct rod (in the example of Fig 1, the central rod) as a consequence of the movement, the more probability it assigns to the movement (see Methods for details).

Once these priors are set, the two strategies use the same probabilistic inference methods introduced before—thus, they only differ for their choice or priors, not the inference. Because they use the same inference methods, the performance of the two strategies, which are based on considerations of plan optimality (“look-ahead”) or perceptual proximity to the goal (“perceptual”) can be directly compared. Furthermore, it is possible to run experiments that consider various (weighted) combinations of look-ahead and perceptual strategies. Combined strategies are created by allocating a percentage of the particles to each of the two strategies during the inference (e.g., 50% of the particles use the priors of the “look-ahead” strategy, and the remaining 50% use the priors of the “perceptual” strategy) while also preventing any resampling during the inference.

Fig 4C illustrates the simulation results for various strategies, which range from a “pure” perceptual strategy (left), where 100% of the particles use the priors of the “perceptual” strategy, to a “pure” look-ahead strategy (right), where 100% of the particles use the priors of the “perceptual” strategy, and all the intermediate cases (e.g., 50% of the particles use the priors of the “perceptual” strategy, and 50% of the particles use the priors of the “look-ahead” strategy). Parameters are reported in Table 1. Our simulations show that the percent of particles able to find the shortest plan from state *S*27 to state *S*20 (i.e., a plan that only includes 7 moves) varies as a function of how many particles of the particle filtering algorithm use the “look-ahead” or “perceptual” strategies. A pure “look-ahead” strategy is the most successful while the performance degrades quickly when increasingly more particles are allocated to the “perceptual” strategy. These results thus speak to an advantage of the “look-ahead” over the “perceptual” strategy, where their differences here are explained in terms of different prior distributions, not of different inferential mechanisms.

**Fig 5:**
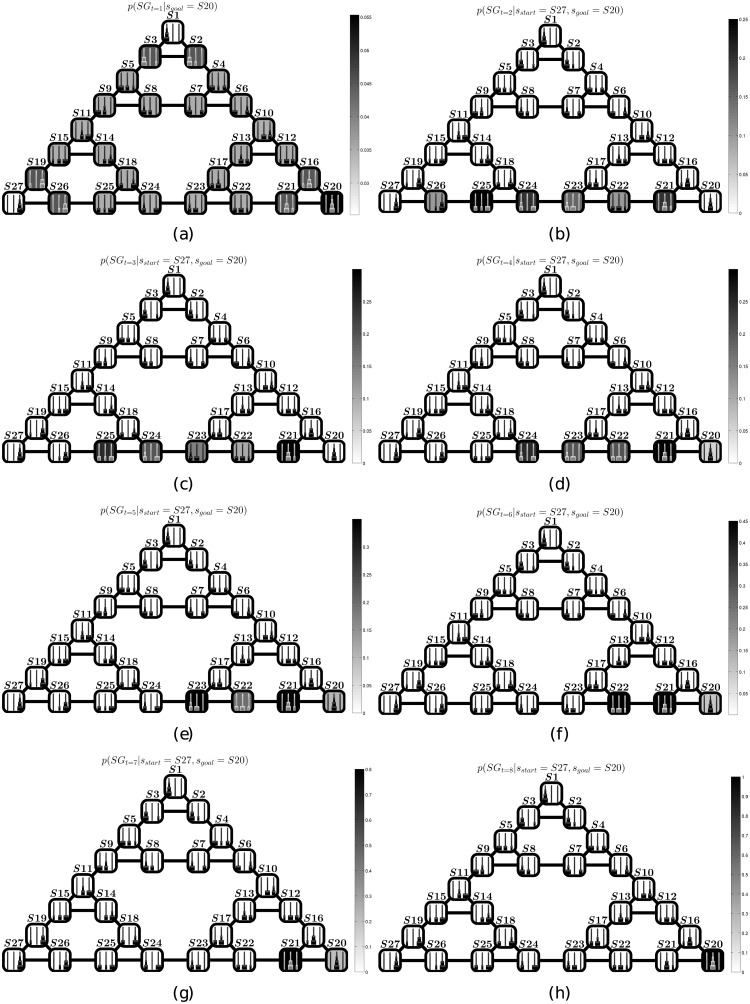
Behavior of a representative simulation using the “look-ahead” strategy. Panels (a-h) show how the *SG* probability distribution varies at each time step (respectively 1–8), during the most frequent solution of a ToH problem starting from state *S*27 and reaching the goal state *S*20, using the “look-ahead” strategy. Note that the solution requires 7 inferential steps, and all the states having high probability lie along the best path from *S*27 to *S*20.

The dynamics of the subgoal probability distributions *p*(*SG*) of the “look-ahead” strategy are shown in Fig 5. The first panel shows the prior *SG* distribution once the start and goal states are set. The successive panels show how this distribution is updated during the inference, reflecting the fact that the particles are approaching the goal location. Effectively, already from the second panel all the high-probability subgoals lie in the best path from the start to the goal. Furthermore, it is evident that during the inference, the “target” candidate subgoals are increasingly closer to the goal location—and the goal location is the highest probability location (only) in the last panel. In other words, this algorithm implicitly creates a “moving target” or “gradient” of intermediate subgoals that permit splitting the problem into more manageable subproblems, carving the huge search space (∼ 10^12^ possible policies).

**Fig 6:**
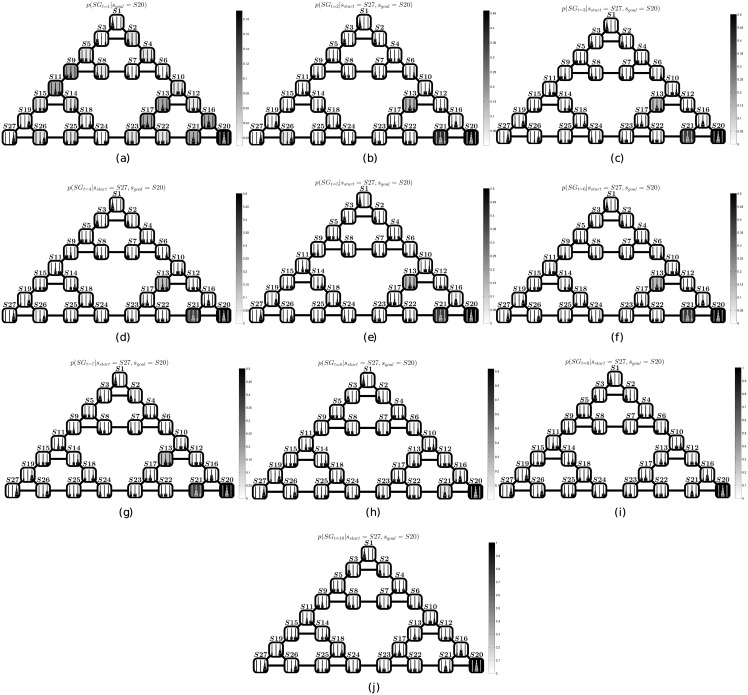
Behavior of a representative simulation using the “perceptual” strategy. Panels (a-j) show how the *SG* probability distribution varies at each time step (respectively 1–10) during the most frequent solution of a ToH problem starting from state *S*27 and reaching the goal state *S*20, using the “perceptual” strategy. Note that, at difference with Fig 5, here the solution requires 9 inferential steps. Furthermore, several of the high probability states (e.g., *S*13, whose activation remains quite high up to the 6th step) lie far away from the optimal path from *S*27 to *S*20. The reason is that these states are perceptually similar to the goal state.

This situation can be contrasted with the dynamics of the subgoal probability distributions *p*(*SG*) of the “perceptual” strategy, shown in Fig 6, which lacks an equivalent gradient. Different from the “look-ahead” strategy, the “perceptual strategy” appears to be impaired by strong priors close to the final goal. It is this bias that produces a myopic strategy and prevents subjects from performing counterintuitive moves that would apparently move farther from the goal state.

**Fig 7:**
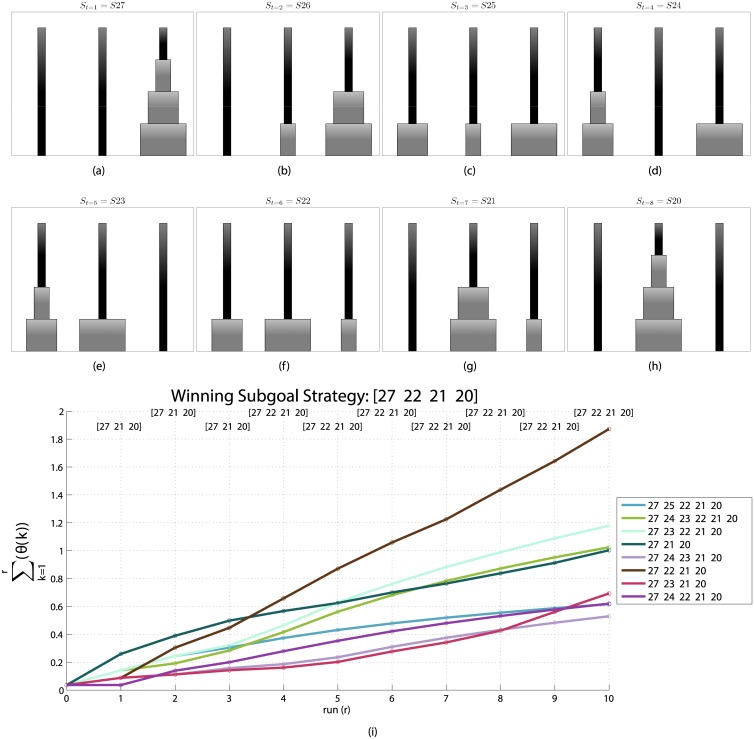
Solution found with higher probability by the look-ahead strategy. (a-h) The *S* states of the *most voted solution* at each time step for the Tower of Hanoi problem starting from state *S*27 and reaching the goal state *S*20. (i) How the winning solution shown in (a-h) is selected based on a competition between different subgoal sequences. At each step of resampling new particles are produced on the specific goal and by the voting mechanism the most successful subgoal-sequence strategy is selected. Notice that several different sequences get votes: in this plot, for the sake of clarity, we only show those sequences that surpass a threshold of 0.4.

These differences between the two strategies have significant effects on their relative performance. The most frequent solution found by the particles using the look-ahead strategy requires 7 inferential steps while the most frequent solution of the perceptual strategy requires 9 inferential steps (to understand how the most frequent or “most voted” solution is computed, see the Method section). The 7-steps solution found with higher probability by the look-ahead strategy is a unique, optimal plan passing through the states: *S*27, *S*26, *S*25, *S*24, *S*23, *S*22, *S*21, *S*20 (which, for illustrative purposes, we also show in the usual Tower of Hanoi format in Fig 7). Note that as shown in Fig 4D most of the particles that find the aforementioned 7-step optimal plan use 2 or 3 subgoals, which highlight an advantage of subgoal-based strategies over strategies that try to solve a problem from start to end without splitting the problem space.

### Empirical support for the analysis of counterintuitive movements

To validate our approach, we used our model to reproduce the results of an empirical study that investigated how adult patients with lesions of the prefrontal lobe and a control group solve Tower of Hanoi puzzles of increasing difficulty [77], see also [67]. The study revealed specific deficits in lesioned patients in resolving a goal-subgoal conflict, in particular in the case of counterintuitive movements requiring to move away (in a perceptual sense) from the goal state.

Here we simulated three ToH problems analogous to those reported in [77], which exemplify an easy, an intermediate, and a challenging situation. All the three problems (P1–P3) had the same goal state *S*20. In the easiest problem P1, the initial state was *S*13. This problem has a simple solution requiring only 3 moves. In the intermediate problem P2, the starting state is *S*27. The shortest solution of this problem requires 7 moves (the maximum number in our Hanoi Tower) and crosses a bottleneck between two community structures. In the hardest problem P3, the starting state is *S*9. Like the intermediate problem, the shortest solution to this problem requires 7 moves and crosses one bottleneck (*S*6 − *S*10). However, this path is harder to find for the presence of more counterintuitive moves, which hinder particularly the perceptual strategy. For example, an agent following the perceptual strategy will tend to do a transition from *S*9 to *S*11, not *S*8, because *S*11 is perceptually closer to the goal state *S*20. This tendency can prevent the agent from finding the optimal path, which passes through *S*8.

In keeping with our previous arguments, we associated the behavior of lesioned patients vs. control group to a different choice of strategies, the former more “perceptual” and the latter more “look-ahead”. Importantly, we do not consider these two strategies to be computationally different (as commonly assumed in the ToH literature); rather, we model both using our inferential method, but using two different prior distributions for subgoals (perceptual priors vs. algorithmic priors).

Accordingly, we associate the behavior of lesioned patients vs. control group to two models. In the former model, corresponding to lesioned patients, 85% of the particles are initialized according to the perceptual prior and 15% are initialized according to the algorithmic prior. in the latter model, corresponding to the control group, 85% of the particles are initialized according to the algorithmic prior and 15% are initialized according to the perceptual prior.

**Fig 8:**
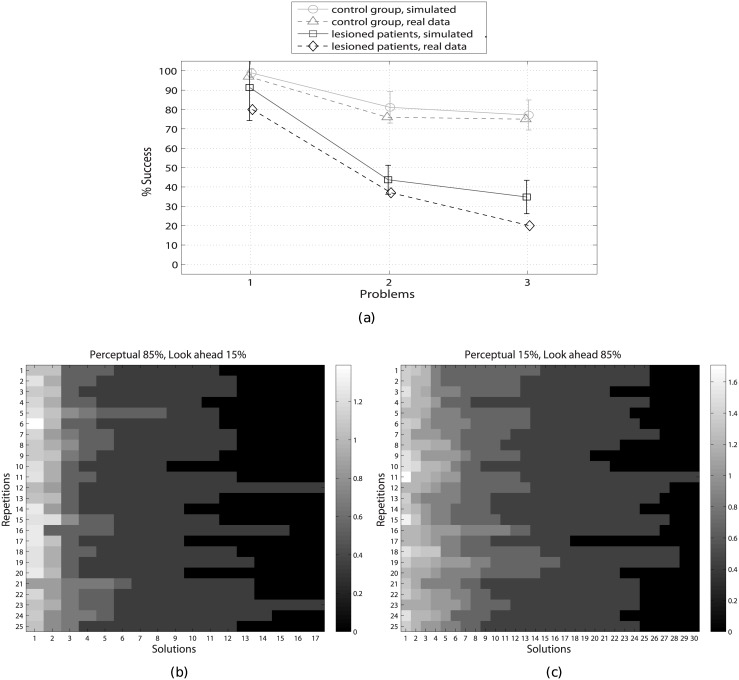
Comparison of real and simulated data on the ToH. (a) Success rate mean and variance, “lesioned patients” and “control subjects” (as reported in [67, 77]) and their simulated equivalents. Results are for 25 simulations. (b-c) Matrices of solutions of 25 simulations of lesioned patients (particle percent: *perceptual* = 85 and *look-ahead* = 15) and control group (particle percent: *perceptual* = 15 and *look-ahead* = 85), respectively. The greyscale indicates the number of votes received by the solutions, i.e., the sum of *θ* values of Eq 13; the lighter the grey, the higher the number of votes, the better the result. The *x*-axis shows that the number of solutions found to the problem P3 by the control group (c) is greater than the number of solutions found by the lesioned patients (b). The possibility to find more solutions suggests a greater flexibility of the look-ahead strategy, which in turn might explain its better performance. Furthermore, it is possible to appreciate that the most voted solutions of the control group receive more votes than the most voted solutions of the lesioned patients (compare their greyscale), indicating a faster decision making process.

Fig 8A shows the results of the experiment (25 executions of each model). As in the human experiment, we measure success rate as the percentage of participants (or executions of the model) that found the shortest path solution to the ToH problem. (Note that in the human experiments, participants solve the experiment only once.)

In P1, the success rate is high for both groups, but the variability of the solutions is higher for lesioned patients. In P2 and P3, the success rate of both groups decreases, but—crucially—it does so more steeply for the simulated lesioned patients, reflecting their difficulties in facing challenging problems that include counterintuitive moves.

Our simulations replicate the pattern of results reported in the empirical study [77], and in particular the steeper decrease in the performance of lesioned patients when the problems require executing counterintuitive movements. This pattern of results cannot be explained by an algorithm that simply introduces noise in the best strategy, but results from different qualitative strategies, with the perceptual strategy that performs quite well in simple cases but not in the more complex ones that include counterintuitive movements. The slightly lower (overall) performance of real participants compared to our simulations might result from minor differences in the set up. Indeed, the three problems P1–P3 reported here correspond to the three problems P5, P1 and P8 in [67, 77], after a mapping between the ToH problem space used in our simulations (with three disks and three pegs) and the slightly more challenging one used in the empirical report (with five disks and three pegs). It is important to note that despite the minor differences in the set up, the structure of the problem is the same (e.g. the number of bottlenecks and counterintuitive moves) and the overall pattern of results of the simulations coherent with the real data.

Besides replicating the pattern of results in [77], our simulations can provide a more fine-grained analysis of the behavior of the two groups. The two matrices of Fig 8B and 8C show the number of solutions to problem P3 (*x*-axis) and relative number of “votes” (grey scale) found by the simulated lesioned patients and control group, respectively. It is possible to appreciate that the simulated control group is able to find many more solutions than the simulated lesioned patients. This result confirms that the look-ahead strategy is more flexible and permits agents to find a broad spectrum of solutions, rather than narrowing the problem space. This increased flexibility is especially advantageous in challenging problems like P3 and might explain the large gap in the performance between the two models, see Fig 8A. Furthermore, it is worth noting that the most voted solutions of the control group receive more votes than the most voted solutions of the lesioned patients (compare the grayscale of Fig 8B and 8C), which in turn implies a faster convergence of the algorithm in the former case.

## Discussion

We have presented a formal approach to human-level problem solving, here exemplified in the Tower of Hanoi (ToH) task. We use probabilistic inference methods that are increasingly adopted to study multiple cognitive domains, such as perception, action and learning [17, 18], supporting the idea that the computations underlying problem solving might share common principles with them. Specifically, we leverage on the *planning-as-inference* framework (PAI) and extend it to address problem solving by introducing a crucial additional mechanism: subgoaling. Our emphasis on subgoaling is in keeping with their recognized importance of subgoaling in human problem solving and cognitive architectures [1, 8–13, 57] and with a vast computational literature showing that subgoals can carve the problem space and reduce the computational complexity of problems [14–16, 47, 66, 78].

Our results illustrate that a subgoaling-based probabilistic inference approach can explain key aspects of human problem solving. Our first two studies focused on what structure or *problem space* humans use. Convergent findings indicate that the time to execute a ToH puzzle is proportional to the complexity of the problem, not to the number of steps in a graph. The complexity of the problem can be characterized as a distance in *problem space* between the start and goal configurations, which does not only consider the number of (physical) steps required to solve a problem, but also computational requirements (e.g., the complexity of solutions and associated computational costs), which in turn are influenced by “community structure” of the problem [74, 75]. In the proposed computational approach, an important constituent of the problem space is prior subgoal distribution *p*(*SG*)—or an a-priori probabilistic estimate of the likelihood of traversing a given set of states. The first two studies thus show that the prior subgoal distribution measure can explain two typical (but otherwise puzzling) idiosyncrasies of human problem solving strategies, and their sensitivity to the (community) structure of the problem at hand.

Our third and fourth studies show that a specific and well-documented deficit in human problem solving—the failure to execute counterintuitive movements—can be explained in terms of a mis-identification and mis-use of (good) subgoals within our probabilistic inference scheme. One interesting aspect of our proposal is that it permits to describe two competing strategies for solving the ToH, look-ahead vs. perceptual [57], within a homogeneous probabilistic inference method, without appealing to two segregated mechanisms. In this perspective, when the perceptual strategy is used, the subgoal distribution quickly collapses into a narrowly-focused problem representation, in which the final goal dominates the inference, preventing subjects to carve the problem in useful ways. In this perspective, the strategy a person uses during problem solving (and her errors) might be predicted by looking at her subgoal distribution prior and during the inference. This is a novel empirical prediction that can be potentially tested in the ToH or related puzzles.

It is worth noting that the disadvantages of the perceptual strategy might be partially compensated by the fact that calculating subgoal distributions using a simple “perceptual distance” between states could be less cognitively demanding than updating them according to the look-ahead strategy. In this perspective, the choice of a more accurate but also more cognitive demanding vs. a simpler but inflexible strategy might obey to computational trade-offs [19, 40, 79–81]. If this hypothesis is correct, introducing a cognitive load would shift the balance towards the latter (perceptual) strategy [82, 83]. This prediction remains to be tested in future research.

To summarize, we have shown that problem solving requires the ability to carve the problem space in useful ways, which do not only (or not necessarily) reflect a simple physical distance, but a distance in a subtler “problem space”. In this perspective, we have shown the advantage of representing possible subgoals in terms of algorithmic priors rather than in terms of a mere perceptual distance from the goal state.

The way we calculate algorithmic priors *P*(*SG*) shares some similarities, but also differences, with alternative ways that have been proposed in the literature that are based on graph theory [74, 75, 84] and information theory [43–45, 85, 86]. In our approach, the algorithmic prior of a state considers, first, the number of policies that generate programs terminating in the state (the more the policies, the higher the probability) and second, the length of these programs (the shortest the programs, the higher the probability)—thus reflecting a prior preference for traversing every state by using the best (shorter) program. In the ToH maze, this second aspect of the algorithm tends to assign higher probability to states that are close to (but are not) vertexes, because the programs that start from vertexes are shorter in these states compared to all the other states, including bottlenecks (mazes with different topologies will have different *p*(*SG*) distributions, of course). This implies that bottlenecks do not have the highest prior probability. This might seem in contrast with a graph-theoretic perspective, where bottlenecks are usually identified as salient structural aspects of the problem [74, 75, 84]. However, two points are in order. First, graph-theoretic algorithms do not assign a probability distribution over all states, but only identify bottlenecks, thus it is hard to directly compare the two methods. Second, in our approach *p*(*SG*) reflects algorithmic probability measures and the prior propensity to visit or traverse a state rather than its perceptual salience or connectedness; and we have shown that this choice of priors permits to model accurately human behavior, especially the preference for paths that include lesser (or lower-rank) bottlenecks. It is possible that graph-theoretic measures and our approach based on algorithmic probability reflect distinct (not necessarily divergent) structural aspects of the problem, the former more revelatory of topological aspects of the maze and oriented towards (optimal) task decomposition [74], and the latter more related to informational constraints of the problem to be solved—to which humans seem to be particularly sensitive. Supporting this idea is the fact that our approach is largely convergent with other methods that identify subgoals using info-theoretic measures such as the *information bottleneck method* [85, 86] and *relevant goal information (RGI)* [43–45]. The RGI method uses the mutual information between goal and action to assign states an information value; like our approach, this method identifies transition points, and assigns bottlenecks low (not high) information value, and high information value to bottleneck neighbors. While using different information measures, the RGI method is largely convergent with our approach as it reflects informational constraints of the problem (e.g., the amount of information an agent needs to maintain about its goal and the points where she needs to change subgoals) that—we argue—are important determinants of human problem solving in challenging tasks such as the ToH.

The methods we use are also related to Hierarchical Reinforcement Learning (HRL) [48]. In particular, the transition *p*(*π*|*s*, *sg*) is related to the concept of an “Option” in HRL [48] but it is expressed probabilistically. In HRL, learned Options influence policy selection and guide the agent transitions for an extended period of time, usually up to a predefined subgoal (e.g., an Option might correspond to “move until the next door”). Rather, here there are no predefined or “cached” subgoal-and-policy pairs. Policies are sampled at each step of the inference, while subgoals are sampled whenever the previously selected subgoal has been reached. Thus, in a sense, this system forms an Option-like structure on-the-fly that guides the agent’s transitions up to the next predefined subgoal. In principle, the (best) Option-like structures (e.g., *p*(*π*|*s*, *sg*)) formed during the inference might be “cached” to facilitate future inferences; this is something we plan to explore in future studies.

## Conclusions

We presented a novel computational theory of human problem solving that is based on probabilistic inference augmented with a subgoaling mechanism. Probabilistic inference methods are increasingly used to explain a variety of cognitive, perceptual and motor tasks, including goal-directed decisions and planning [17, 23, 30, 34, 37, 38, 87]. Here we show that probabilistic inference, when enhanced with a subgoaling mechanism, can explain various aspects of human problem solving, too, including its idiosyncrasies and deficits, such as the human sensitivity to the structure of the problem space, and patient deficits in handling counterintuitive moves and goal-subgoal conflicts. We focused on the well-studied Tower of Hanoi (ToH) task, which has been modeled using several computational frameworks such as the cognitive architecture ACT-R [88] and various symbolic [81, 89] and subsymbolic systems [90], none of which however use the principles of probabilistic inference proposed here.

This computational analysis suggests that human problem solving does not necessarily need to be considered a special(ized) domain or module of cognition, but could use the same probabilistic computations that are widely studied in other fields of cognitive science. Along similar lines, it is not necessary to assume that suboptimal strategies such as the perceptual strategy are mechanistically different from the optimal solution or heuristics. Our study shows that they can be explained within the probabilistic framework introduced here under the assumption of a different problem (prior) representation.

This computational analysis underlies the importance of subgoaling in problem solving, too, showing that subgoals—as expressed for example in the prior subgoal distribution *p*(*SG*)—permit to carve the problem space in useful ways. Importantly, subgoals define a metric for the problem that is sensitive to the probability of transitions between states rather than to the mere count of the number of states from start to goal. Humans are sensitive to key aspects of this metric, such as its community structure [74]. Furthermore, subgoals can be used as “waypoints” that permit finding parsimonious solutions: the algorithm splits the problem into smaller subproblems, each requiring less information to be encoded compared to a solution from start to end (this adds on to the fact that the inferential method tends to select programs having higher algorithmic probability and thus having a shorter code-length [65, 66, 68, 69]). From a cognitive perspective, the overall *divide et impera* strategy can be less taxing for an agent, because she only needs to “remember” a part of the solution (e.g., the path to the next subgoal) at every moment in time. Reaching a subgoal (as signaled by the node *F* in our model) marks a transition to a next subgoal, and implies that the agent can “forget” past information [43–45]. Furthermore, finding waypoints permits to learn and store subroutines (e.g. Options in HRL) that—in principle—can be reused in future planning problems, thus saving resources [91–93]. Finally, the failure to use subgoal information in appropriate ways can explain specific problem solving deficits such as the inability to execute *counterintuitive movements* of prefrontal patients [57], without appealing to separate cognitive mechanisms for look-ahead vs. perceptual strategies.
